# “Young at heart”: Regenerative potential linked to immature cardiac phenotypes

**DOI:** 10.1016/j.yjmcc.2016.01.026

**Published:** 2016-03

**Authors:** Renata S.M. Gomes, Philipp Skroblin, Alex B. Munster, Hannah Tomlins, Sarah R. Langley, Anna Zampetaki, Xiaoke Yin, Fiona C. Wardle, Manuel Mayr

**Affiliations:** a King's British Heart Foundation Centre, King's College London, London, UK; b Cardiovascular Development, Randall Division, King's College London, UK

**Keywords:** DIGE, difference in-gel electrophoresis, MI, myocardial infarction, LC-MS/MS, liquid chromatography tandem mass spectrometry, Regeneration, Stem cell, Myofilament, Cardiomyocyte, Proteomics

## Abstract

The adult human myocardium is incapable of regeneration; yet, the zebrafish (*Danio rerio*) can regenerate damaged myocardium. Similar to the zebrafish heart, hearts of neonatal, but not adult mice are capable of myocardial regeneration. We performed a proteomics analysis of adult zebrafish hearts and compared their protein expression profile to hearts from neonatal and adult mice. Using difference in-gel electrophoresis (DIGE), there was little overlap between the proteome from adult mouse (> 8 weeks old) and adult zebrafish (18 months old) hearts. Similarly, there was a significant degree of mismatch between the protein expression in neonatal and adult mouse hearts. Enrichment analysis of the selected proteins revealed over-expression of DNA synthesis-related proteins in the cardiac proteome of the adult zebrafish heart similar to neonatal and 4 days old mice, whereas in hearts of adult mice there was a mitochondria-related predominance in protein expression. Importantly, we noted pronounced differences in the myofilament composition: the adult zebrafish heart lacks many of the myofilament proteins of differentiated adult cardiomyocytes such as the ventricular isoforms of myosin light chains and nebulette. Instead, troponin I and myozenin 1 were expressed as skeletal isoforms rather than cardiac isoforms. The relative immaturity of the adult zebrafish heart was further supported by cardiac microRNA data. Our assessment of zebrafish and mammalian hearts challenges the assertions on the translational potential of cardiac regeneration in the zebrafish model. The immature myofilament composition of the fish heart may explain why adult mouse and human cardiomyocytes lack this endogenous repair mechanism.

## Highlights

• Proteomics reveals minimal overlap between adult mouse and adult zebrafish hearts.
• Gene expression analysis confirms profound differences in myofilament composition.
• The adult zebrafish heart is more similar to a newborn mouse heart.
• The relative immaturity is further supported by cardiac microRNA data.

## Graphical abstract

**Figure:**
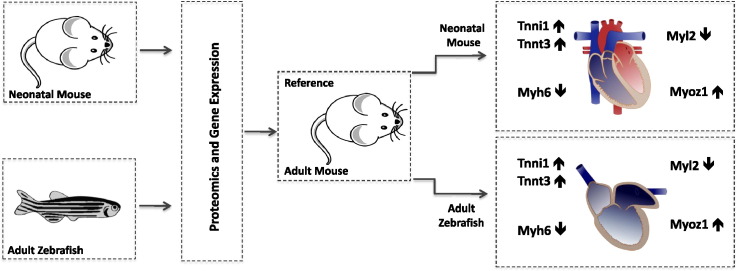

## 1 Introduction

The zebrafish heart has been extensively studied in order to understand its cardioregenerative mechanisms with the hope of translating these findings to humans. The adult zebrafish heart is able to regenerate within 130 days after having 20–30% of its ventricle damaged by cryo-injury [1]. Genetic ablation of myocytes produces a loss of around 60%, the full extent of which is replaced within 30 days. The large discrepancy in regeneration time is explained by the fact that cryo-injury results in the formation of a large fibrotic area, whereas with genetic ablation no collagen deposition occurs (only a small fibrin clot is observed) [2]. After myocardial infarction (MI) in humans, the formation of scar tissue is an irreversible event. However, neonatal mice are capable of myocardial regeneration: upon ventricular apex amputation, 1-day-old mice display full tissue regeneration not dissimilar in manner to the zebrafish [3]. The neonatal mouse heart will also regenerate after induction of MI via left anterior descending coronary artery ligation. Nonetheless, 7 days after parturition mice lose this ability to regenerate damaged myocardium.

Multiple studies have examined the proteomic profile of the zebrafish [4], [5], yet no comparisons have been attempted with mammals such as mice. In this study we investigate the similarities of the zebrafish heart with the postnatal and adult mouse heart.

## 2 Materials and methods

### 2.1 Mouse hearts

Mice, C57BL/6 were maintained under standard housing, all procedures performed had the necessary UK Home Office and local ethical approval for animal procedures. Mating pairs were set up to attain hearts over specific time points from birth. Hearts were excised from newborn pups (within hours of birth), 4 and 14 days post birth and during adulthood (8–16 weeks old). Hearts were removed, cleared of connective tissue, washed thoroughly in ice-cold PBS, frozen immediately on dry ice and stored at − 80 °C until processing.

### 2.2 Zebrafish hearts

Wild-type AB strain zebrafish (*Danio rerio*) aged 14–18 months were euthanized in 0.16% Tricaine (MS-222, Sigma Aldrich). Hearts were surgically dissected and any connective tissue removed. The dissected hearts were washed thoroughly in ice-cold PBS to remove contaminants or debris, frozen immediately on dry ice and stored at − 80 °C.

### 2.3 RNA extraction, reverse transcription and quantitative real-time PCR

RNA extraction, reverse transcription and quantitative real-time PCR (qRT-PCR) was performed as detailed in extended methods.

### 2.4 Protein extraction and immunoblot analysis

Hearts were weighed and powderised in liquid nitrogen and crushed using a mortar and pestle. Ice-cold lysis buffer (100 mM Tris–HCl pH 7.4, 1% Triton X-100, protease and phosphatase inhibitors (Sigma, UK)), at a ratio of 100 μl per 10 mg of tissue was added. Samples were centrifuged at 14,000*g* for 10 min at 4 °C. The soluble and non-soluble fractions were separated and added to Laemmli buffer after quantification of protein concentration using the BCA assay (Thermo Scientific, USA). Immunoblots were performed as described in extended methods section.

### 2.5 Difference in-gel electrophoresis (DIGE) and liquid chromatography tandem mass spectrometry (LC-MS/MS)

Proteomics analysis was performed as previously described [6]. A detailed protocol is provided online at http://www.vascular-proteomics.com/.

## 3 Results and discussion

### 3.1 Proteomics analysis of mouse and zebrafish hearts

**Fig. 1:**
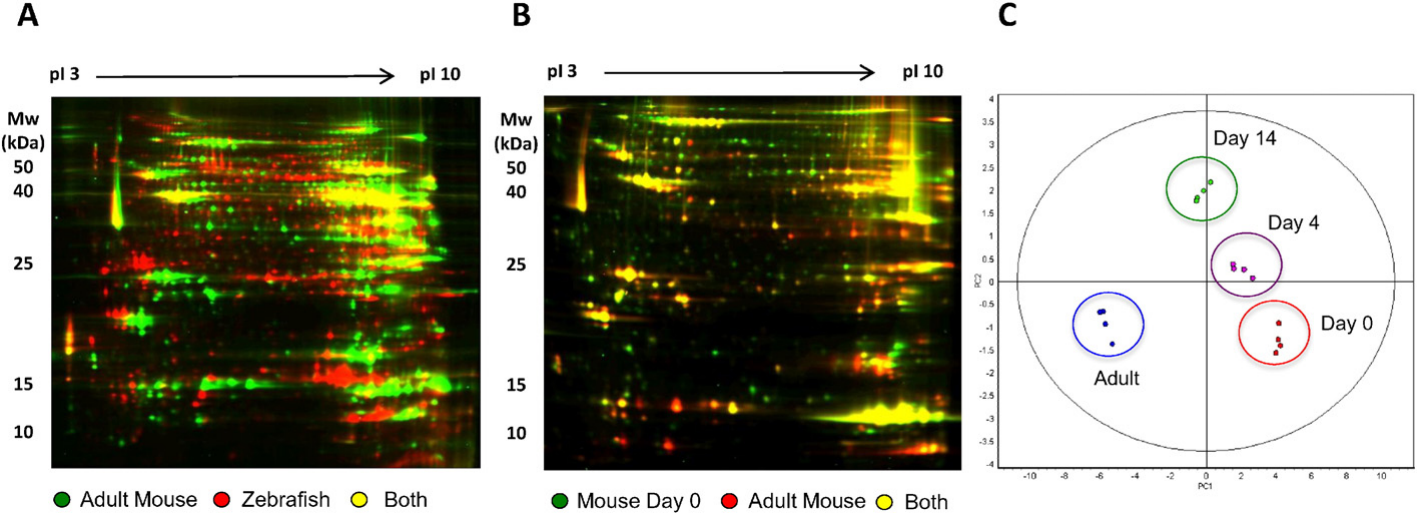
DIGE comparison of the cardiac proteome. A) Overlay of adult zebrafish (red) and adult mouse (green) where overlapping proteins are seen by yellow colour. Note that there are few common protein spots between the adult mouse and adult zebrafish heart. B) Adult mouse (red) and neonatal mouse (day 0, green) heart protein overlay where overlapping proteins are again yellow. C) Principle component analysis. Analysis of differential protein expression on neonatal (day 0, red), day 4 (pink), day 14 (2 weeks, green) and adult mouse hearts (blue). The protein expression among the groups is clearly differentiated.

Hearts of neonatal and adult mice (0, 4, 14 days and 8–16 weeks) and adult zebrafish (18 months old) were dissected and processed for DIGE and LC–MS/MS analysis (Fig. 1). The cardiac proteome from adult mice and adult zebrafish was notably different (Fig. 1.A, Supplementary Fig. 1A, Supplementary Tables 2–3). Similarly, there were differences between the proteomes of neonatal and adult mouse hearts, yet these changes were less pronounced (Fig. 1B). From the latter DIGE gels, 151 spots were excised for identification by LC-MS/MS (p value < 0.05, fold change ≥ 1.5) (Supplementary Fig. 1B and Supplementary Table 4). The principle component analysis of the proteome of postnatal and adult mouse hearts showed clear separation between age groups (Fig. 1C). Enrichment analysis of the selected proteins revealed an over-representation of DNA synthesis-related systems in the adult zebrafish proteome, whereas for adult mice there was mitochondria-related protein predominance (Supplementary Fig. 2).

### 3.2 Validation by immunoblotting

It was apparent that several myofilament protein changes characteristic of the adult mouse heart were absent in adult zebrafish hearts. Proteomic analysis of mouse hearts revealed age-dependent differences in troponin T (Tnnt), troponin I (Tnni), myosin light chain (Myl), myosin heavy chain (Myh), myozenin-2 (Myoz2) and nebulette expression. Although most antibodies are not validated for zebrafish, immunoblot analyses were attempted for several differentially expressed proteins on all mouse samples alongside zebrafish hearts: myozenin-2, nebulette and troponin-T (Supplemental Fig. 3).

### 3.3 Gene expression of myofilament proteins

**Fig. 2:**
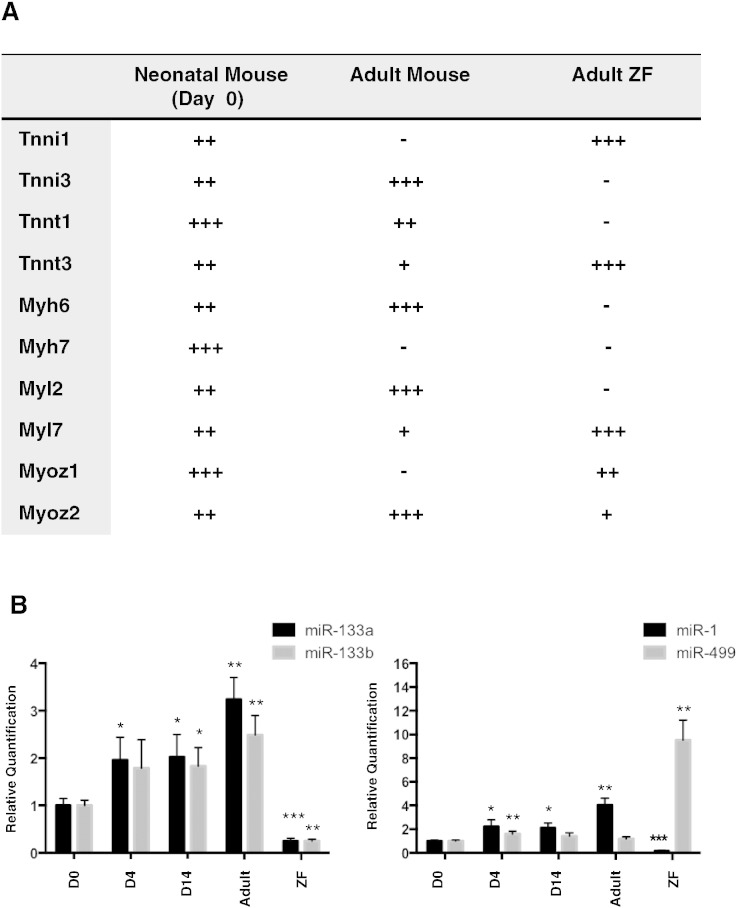
Comparison by qPCR. A) Summary of qPCR data for myofilament expression. The relative abundance was ranked from lowest to highest expression (+, ++, +++) in neonatal mouse, adult mouse and adult zebrafish (ZF) hearts. The absence of expression or very low levels was denoted by (−). Details are shown in Supplementary Figure 4. B) Measurements of cardiac miRNAs in the postnatal (day 0, day 4, day 14) and adult mouse heart and the adult ZF heart. Expression relative to internal control. Comparisons of gene expression to day 0 (D0) were performed using Student's t-tests. * denotes p ≤ 0.05; ** p ≤ 0.01; *** p ≤ 0.001.

Due to the uncertain reliability of antibodies for zebrafish proteins, we performed qRT-PCR of myofilament protein-encoding genes (summary of data as Fig. 2A). Nebulette was undetectable in zebrafish hearts, consistent with the immunoblot data.

Three isoforms of Troponin T (*Tnnt*) are expressed in neonatal mouse hearts: *Tnnt1* (slow skeletal), *Tnnt2* (cardiac) and *Tnnt3* (fast skeletal). Cardiac *Tnnt2* is expressed constitutively, whereas the levels of *Tnnt1* and *Tnnt3* decrease throughout mouse development. In contrast to mice, *tnnt3b* is the predominant isoform in adult zebrafish, which is found at the highest levels within immature hearts and serves as a marker for the switch between the foetal/neonatal and the adult heart [7] (Supplementary Fig. 4A).

In the case of Troponin I, there are also three mammalian isoforms: Tnni1 (slow skeletal), Tnni2 (fast skeletal) and Tnni3 (cardiac). All are expressed in neonatal mouse. *Tnni1* expression declines throughout postnatal development and is absent from adult hearts whereas levels of *Tnni3* increase over time. In adult zebrafish hearts, *tnni3*, the cardiac specific isoform, was not detectable while high levels of the slow-skeletal isoform, *tnni1b*, were found (Supplementary Fig. 4B).

Next we analysed the myosin heavy and light chains. For the myosin heavy chains, *Myh7b* shows no significant changes, whereas *Myh6* significantly increases and *Myh7* decreases in mice over time. In zebrafish hearts, *myh7b* was detectable (Supplementary Fig. 4C) unlike *myh6* or *myh7*. Instead, a ventricular myosin heavy chain-like protein was identified by proteomics (Supplementary Table 3, spots R18 and R19). For the light chains, *Myl7* decreases as the ventricular isoforms *Myl2* and *Myl3* increase after birth. In zebrafish, *myl2* was not detectable whereas high levels of *myl7* were found (Supplementary Fig. 4D).

Finally, mammals express three isoforms of myozenin: *Myoz1* (myozenin-1, calsarcin-2), *Myoz2* (myozenin-2, calsarcin-1) and *Myoz3* (myozenin-3, calsarcin-3). All three isoforms are expressed in neonatal mouse hearts. The expression of *Myoz1* and *Myoz3* is lost during postnatal cardiac development but *Myoz2* levels increase over time. MYOZ2 is a calcineurin-interacting protein, which tethers calcineurin to α-actinin at the z-line of the sarcomere of cardiac tissue. Adult zebrafish hearts express the cardiac isoform *myoz2* but, in contrast to adult mouse hearts, they also express *myoz1*, the skeletal isoform [8] that is expressed in the neonatal mouse heart (Supplementary Fig. 4E).

### 3.4 Cardiac microRNAs

In addition to myofilament proteins, the expression of four microRNAs (miRNA, miR) involved in cardiac development and function was analysed (Fig. 2B): miR-133a and b, miR-1 and miR-499. Two other important cardiac miRNAs, *mir-208a* and *mir-208b*, do not exist in zebrafish. MiR-133a and b (a regulator of myocyte enhancer protein 2) and miR-1 (important in myoblast to myotube differentiation) significantly increased over time in developing mouse hearts yet were present at very low levels in the adult zebrafish heart. MiR-499, which is highly expressed in the late stages of cardiac differentiation [9], was significantly up-regulated at day 4 and 14 post birth compared to adult mouse hearts. Unlike other cardiac miRs measured, *miR-499* expression was higher in zebrafish cardiac tissue compared to mice. The zebrafish genome contains 3 loci encoding *miR-499* in contrast to a single mammalian locus [10]. MiR-499 plays a key role in inhibition of cardiomyocyte apoptosis through its suppression of calcineurin-mediated dephosphorylation of dynamin-related protein-1 [11] and has been implicated in myocardial regeneration [12].

## 4 Conclusion

In the present study, we applied proteomics to compare the protein content of the zebrafish hearts to neonatal and adult mouse hearts. We observed remarkable differences in the myofilament composition that question the applicability of findings in zebrafish to mammalian hearts. The profound differences in structural gene expression place the (regenerative) zebrafish heart rather in the vicinity of the (proliferative) neonatal, but not the adult mouse hearts. Proteomics proved to be particularly useful in this study since only few validated antibodies for zebrafish are currently available. Thus, a comprehensive analysis at the protein level could not have been performed by other means than mass spectrometry [4], [5]. The immaturity of the zebrafish heart was further confirmed by gene expression analysis of myofilament proteins and cardiac miRNAs. It is therefore questionable if promitotic stimuli that drive cardiac regeneration in zebrafish may be capable of inducing cardiac regeneration in adult mammalian cardiomyocytes. The zebrafish has a highly trabeculated heart with thin chamber walls containing a low number of cardiac fibroblasts, rendering it a less rigid matrix with ease for remodelling. The internal pressure of 2.5 mmHg within the fish heart is very low compared to the mammalian heart [13], and it is also extremely resistant to hypoxia and adapts to ischemia readily. Compared to zebrafish hearts, adult mammalian cardiomyocytes represent an entirely different substrate that have become unresponsive to promitotic stimuli after birth probably due to the transition from a hypoxic to a hyperoxic environment [14] and the increased mechanical load [15].

## Disclosures

None declared.
