# Supplementary material for: “Young at heart”: Regenerative potential linked to immature cardiac phenotypes
Source: J Mol Cell Cardiol. 2016 Mar;92:105–8. doi: 10.1016/j.yjmcc.2016.01.026 (PMC4796039; doi:10.1016/j.yjmcc.2016.01.026)

SUPPLEMENTAL MATERIAL

"Young at Heart":

Regenerative Potential Linked to Immature Cardiac Phenotypes

Renata S.M. Gomes[1], Philipp Skroblin[1], Alex B. Munster[1], Hannah Tomlins[1], Sarah R. Langley[1], Anna Zampetaki[1], Xiaoke Yin[1], Fiona Wardle[2], Manuel Mayr[1*]

[1] King's British Heart Foundation Centre, King's College London, London, UK;

[2] Cardiovascular Development, Randall Division, King's College London, UK.

Corresponding author:

Prof. Manuel Mayr, King's British Heart Foundation Centre, King's College London, 125 Coldharbour Lane, London SE5 9NU, UK.

E-mail address: manuel.mayr@kcl.ac.uk

Extended Methods

RNA extraction, reverse transcription and quantitative real-time PCR

Single mouse hearts or pools of two zebrafish hearts were homogenized in QIAzol Lysis reagent using FastPrep Tubes, Lysing Matrix D (MP Biomedicals Europe, France) and total RNA was subsequently isolated with the miRNeasy mini RNA extraction kit (QIAgen, Germany) (1). For gene expression analysis, 200ng RNA was reverse transcribed using the VILO Superscript cDNA synthesis kit (Life Technologies). The qPCR reaction mix was comprised of 2.5µl TaqMan® Universal PCR Master Mix, No AmpErase® UNG (Life Technologies, UK), 0.25µl of the respective FAM-labelled Taqman assay (Life Technologies, UK), and 2.25µl diluted RT product corresponding to 1ng RNA. MiRNAs (50 ng input material) were reverse transcribed using the TaqMan® MicroRNA Reverse Transcription Kit, Megaplex rodent primers Pool A (Life Technologies, UK). 2.25µl diluted RT product corresponding to 0.225ng RNA were used for qPCR. QPCRs were run on a ViiA7 qPCR machine (Life Technologies, UK) and the ΔΔCt method was used to determine the relative quantification of targets. Taqman assays used for qPCR are listed in **Supplementary Table 1.**

Protein extraction and immunoblot analysis

Hearts were weighed and powdered in liquid nitrogen and crushed using a mortar and pestle. Ice-cold lysis buffer, at a ratio of 100µl per 10mg of tissue was added. The lysis buffer consisted of a combination of 100mM Tris-HCl pH 7.4, 1% Triton X-100, protease and phosphatase inhibitors (Sigma, UK) (2). Samples were centrifuged at 14,000g for 10 minutes at 4 °C. The soluble and non-soluble fractions were separated and added to Laemmli buffer and denatured at 98°C for 5min before being briefly

centrifuged, this after quantification of protein concentration using the BCA assay (Thermo Scientific, USA). 10μg of protein were loaded per lane, separated on 4-12% gradient gels (Life Technologies, UK) at 130-170V for 100-120min in NuPage MOPS SDS Running Buffer (Life Technologies, UK) diluted with distilled water. Subsequently, the samples were transferred to nitrocellulose membranes (GE Healthcare, USA) in ice-cold transfer buffer (25mM Tris Base and 200mM Glycine dissolved in 20% methanol) (3). Next, membranes were blocked in 5% milk in PBS (PBS, Lonza, UK) with 0.1% Tween (PBST) for 1hr on a shaker at room temperature. After a brief wash in PBST, they were incubated overnight with primary antibodies in 5% BSA (Sigma Aldrich, UK) with 0.01% sodium azide (Sigma Aldrich, UK) (Table 2). The following day, after 3 washes in PBST, 15 min each, the membranes were incubated for 1hr at room temperature with the HRP-labelled secondary antibody diluted in 5% milk in PBST, according to the source of the primary antibody. The membranes were washed in PBST 3 times for 15 min and developed using ECL western blotting detection reagent using X-ray films (FUJIFILM, USA). Immunoblots were quantified by densitometry using Image-J software. The following primary antibodies were purchased from Abcam, UK: monoclonal Troponin T (ab130003) and polyclonal nebulette (ab99420) both used at 4 μg/ml. Monoclonal myozenin 2 (sc-377359) from Santa Cruz was used at 1:100 dilution. Immunoreactivity was visualized with horseradish peroxidase-linked rabbit anti-mouse or swine anti-rabbit (DAKO) at 1:2000.

Difference in-gel electrophoresis

The heart tissue was homogenized in lysis buffer (9.5M urea, 2% w/v CHAPS, 1% w/v DTT and 0.8% Pharmalyte pH3-10) with Lysing matrix D (MP Biomedicals, Solon, USA) using FastPrep 120 (Krackeler Scientific Inc, USA) (4). Samples were

further processed using ReadyPrep 2D Cleanup kit (Bio-Rad, UK). The samples were then resuspended in DIGE lysis buffer (8M urea, 4% w/v CHAPS, 30mM TrisHCl, pH 8.5). Protein samples were labelled with Cy3 or Cy5 fluorescent dye (DIGE Fluor minimal dyes, GE Healthcare, USA) and mixed with Cy2-labelled internal standard. Following overnight rehydration, IPG dry strips (pH3-10, nonlinear, 18cm, GE Healthcare, USA) were focused on the IPGphor for 29kVhr. The strips were subsequently reduced in equilibration solution (6M urea, 2% w/v SDS, 30% w/v glycerol, 50mM Tris, pH 8.8) with 10mg/mL DTT for 15min, and alkylated in equilibration solution with 48mg/mL iodoacetamide for another 15min before put on top of large format 12% polyacrylamide gels. SDS-PAGE was performed in Ettan Dalt six electrophoresis system (GE Healthcare, USA) at 30W/gel. The gels were then scanned in Ettan DIGE imager (GE Healthcare, USA) and the images were analysed using DeCyder version 7.0 (GE Healthcare, USA). Differentially expressed spots of interest were manually excised for further MS analysis.

Liquid chromatography tandem mass spectrometry (LC-MS/MS)

Tryptic in-gel digestion was performed overnight using the Investigator ProGest (DigiLab,USA) robotic digestion system (5). The digested peptide solution was dried using a SpeedVac concentrator (Thermo Fisher Scientific, UK) and resuspended in 20µl of 0.1% formic acid. The samples were separated by nano-flow HPLC on a reverse phase column (C18 PepMap100, 3µm, 100 Å, 25cm; Thermo Scientific, USA) before injected to an LTQ Orbitrap XL mass spectrometer (Thermo Scientific, USA). Spectra were collected from the mass analyzer (Orbitrap) using full ion scan mode over the *m/z* range 450-1600 followed by data-dependent MS/MS scans on 6 most abundant ions with dynamic exclusion. Database searches were performed using Mascot version 2.3.01 against the IPI mouse database version 3.87 (59534 entries)

and the IPI zebrafish database version 3.86 (40470 entries), or UniProt/SwissProt mouse database version 2013_08 (16619 entries), with the following parameters: precursor tolerance 10ppm, fragment tolerance 0.8Da, carbamidomethylation on cysteines as fixed modification and oxidation on methionine as variable modification, 2 missed cleavages were allowed. Scaffold software (Version 4.3.2, Proteome Software inc, Portland, OR) was then used to validate MS/MS based peptides and protein identification and assign spectral counts across the individual samples. Protein identifications were accepted if they could be established at greater than 99.0% probability with at least 2 unique peptides.

Statistical and bioinformatic analysis

Principal component analysis (PCA) was used to visualize multidimensional DIGE data using DeCyder version 7.0 (GE Healthcare, USA). Significance of differential transcript or protein expression was assessed using unpaired Student's t-test with unequal variance. Results were given as means±SD. P-values ≤ 0.05 were considered significant.

Online References

1. Abonnenc M, Nabeebaccus AA, Mayr U, Barallobre-Barreiro J, Dong X, Cuello F, et al. Extracellular matrix secretion by cardiac fibroblasts: role of microRNA-29b and microRNA-30c. Circ Res 2013;113:1138-47.
2. Cuello F, Shankar-Hari M, Mayr U, Yin X, Marshall M, Suna G, et al. Redox state of pentraxin 3 as a novel biomarker for resolution of inflammation and survival in sepsis. Mol Cell Proteomics 2014;13:2545-57.
3. Didangelos A, Mayr U, Monaco C, Mayr M. Novel Role of ADAMTS-5 Protein in Proteoglycan Turnover and Lipoprotein Retention in Atherosclerosis. J Biol Chem 2012;287:19341-5.
4. Yin X, Dwyer J, Langley S, Mayr U, Xing Q, Drozdov I, et al. Effects of perhexiline-induced fuel switch on the cardiac proteome and metabolome. J Mol Cell Cardiol 2013;55:27-30.
5. Yin X, Bern M, Xing Q, Ho J, Viner R, Mayr M. Glycoproteomic analysis of the secretome of human endothelial cells. Mol Cell Proteomics 2013;12:956-78.

Supplementary Table 1. Taqman assays used for qPCR.

Gene	Species	Taqman Assay ID
18S	Mouse	Mm03928990_g1
Myh6	Mouse	Mm00440359_m1
Myh7	Mouse	Mm01319006_g1
Myh7b	Mouse	Mm01249941_m1
Myl2	Mouse	Mm00440384_m1
Myl3	Mouse	Mm00803034_m1
Myl7	Mouse	Mm01183005_g1
tnnc1	Mouse	Mm00437115_g1
tnnc2	Mouse	Mm01243775_g1
tnni1	Mouse	Mm00502426_m1
tnni2	Mouse	Mm00437157_g1
tnni3	Mouse	Mm00437164_m1
tnnt1	Mouse	Mm00449089_m1
Tnnt2	Mouse	Mm01290256_m1
Tnnt3	Mouse	Mm01137842_g1
Tnnt3	Mouse	Mm01268863_m1
miR-1	Mouse/Zebrafish	2222
miR-499	Mouse/Zebrafish	1352
miR-133a	Mouse/Zebrafish	2246
miR-133b	Mouse/Zebrafish	2247
18S	Zebrafish	Hs99999901_s1
myh6	Zebrafish	Dr03112899_s1
myl2	Zebrafish	Dr03142386_m1
myl7	Zebrafish	Dr03105697_m1
tnnc1a	Zebrafish	Dr03106128_mH
tnnc1b	Zebrafish	Dr03089208_g1
tnnc2	Zebrafish	Dr03432983_g1
tnni1al	Zebrafish	Dr03146635_m1

tnni1b	Zebrafish	Dr03090193_m1
tnni2a.1	Zebrafish	Dr03102546_m1
tnni2b.1	Zebrafish	Dr03083605_g1
tnnt1	Zebrafish	Dr03201591_s1
tnnt2a	Zebrafish	Dr03131740_g1
tnnt2d	Zebrafish	Dr03090816_g1
tnnt3a	Zebrafish	Dr03150378_g1
tnnt3b	Zebrafish	Dr03086581_m1
vmhc/myh7bb	Zebrafish	Dr03431115_g1
zgc:66286	Zebrafish	Dr03113522_m1

Supplementary Table 2. Identifications of selected protein spots of adult mice hearts.

Spot	Protein name	Accession Number	Mw (Da)	Protein identification probability	#unique peptides	#unique spectra	#total spectra	Percentage sequence coverage
G01	Myosin regulatory light chain 2, ventricular/cardiac muscle isoform	IPI00555015	18,865	100.0%	6	8	15	35.5%
G02	Myosin regulatory light chain 2, ventricular/cardiac muscle isoform	IPI00555015	18,865	100.0%	9	15	28	38.0%
G03	Myosin regulatory light chain 2, ventricular/cardiac muscle isoform	IPI00555015	18,865	100.0%	11	15	24	56.0%
G04	Myosin light chain 3	IPI00133392	22,422	100.0%	7	12	21	55.9%
G05	Myosin light chain 3	IPI00133392	22,422	100.0%	8	16	26	56.9%
G06	Isoform Smooth muscle of Myosin light polypeptide 6	IPI00354819	16,961	100.0%	4	5	8	32.5%
G06	ATP synthase subunit delta, mitochondrial	IPI00453777	17,600	100.0%	3	5	8	17.3%
G07	Cytochrome c oxidase subunit 5A, mitochondrial	IPI00120719	16,102	100.0%	4	4	7	21.9%
G08	Fatty acid-binding protein, heart	IPI00230124	14,819	100.0%	8	11	18	57.9%
G09	NADH dehydrogenase [ubiquinone] iron-sulfur protein 8, mitochondrial	IPI00170093	24,039	100.0%	2	3	4	13.7%
G10	apolipoprotein A-I preproprotein	IPI00877236	30,615	100.0%	6	8	15	20.8%
G11	Isoform 1 of NADH dehydrogenase [ubiquinone] flavoprotein 2, mitochondrial	IPI00169925	27,286	100.0%	6	10	18	35.1%
G12	Thioredoxin-dependent peroxide reductase, mitochondrial	IPI00116192	28,127	100.0%	2	2	3	8.2%
G13	ATP synthase subunit beta, mitochondrial	IPI00468481	56,301	100.0%	7	8	11	16.6%
G14	Isoform 1 of 60 kDa heat shock protein, mitochondrial	IPI00308885	60,957	100.0%	5	6	8	10.6%
G15	Isoform 1 of 60 kDa heat shock protein, mitochondrial	IPI00308885	60,957	100.0%	13	15	26	37.5%
G16	NADH-ubiquinone oxidoreductase 75 kDa subunit, mitochondrial	IPI00308882	79,778	100.0%	21	31	54	33.3%
G17	NADH-ubiquinone oxidoreductase 75 kDa subunit, mitochondrial	IPI00308882	79,778	100.0%	20	28	51	30.1%
G18	Stress-70 protein, mitochondrial	IPI00133903	73,529	100.0%	10	11	18	20.3%
G18	Heat shock cognate 71 kDa protein	IPI00323357	70,873	100.0%	9	12	18	18.4%
G19	Stress-70 protein, mitochondrial	IPI00133903	73,529	100.0%	11	13	23	18.9%
G20	Stress-70 protein, mitochondrial	IPI00133903	73,529	100.0%	2	2	3	4.3%
G20	Heat shock cognate 71 kDa protein	IPI00323357	70,873	100.0%	2	2	3	2.9%
G21	Serum albumin	IPI00131695	68,693	100.0%	14	15	23	20.4%
G22	Dihydrolipoyllysine-residue acetyltransferase component of pyruvate dehydrogenase complex, mitochondrial	IPI00153660	67,943	100.0%	6	10	18	10.4%
G23	Serotransferrin	IPI00139788	76,724	100.0%	6	9	15	12.2%
G24	Serotransferrin	IPI00139788	76,724	100.0%	16	21	37	31.1%
G25	Serotransferrin	IPI00139788	76,724	100.0%	14	15	26	24.0%
G26	Aconitate hydratase, mitochondrial	IPI00116074	85,466	100.0%	24	33	58	32.4%
G27	Succinate dehydrogenase [ubiquinone] flavoprotein subunit, mitochondrial	IPI00230351	72,586	100.0%	18	22	35	31.6%
G28	Unidentified	-	-	-	-	-	-	-
G29	Dihydrolipoyl dehydrogenase, mitochondrial	IPI00874456	54,599	100.0%	3	3	3	5.7%
G29	Isoform M1 of Pyruvate kinase isozymes M1/M2	IPI00845840	57,986	100.0%	2	2	4	3.4%
G30	Isoform M1 of Pyruvate kinase isozymes M1/M2	IPI00845840	57,986	100.0%	14	18	34	35.2%
G31	Trifunctional enzyme subunit beta, mitochondrial	IPI00115607	51,388	100.0%	18	23	37	37.9%
G32	Alpha-enolase	IPI00462072	47,142	100.0%	6	8	17	27.6%
G33	Beta-enolase	IPI00228548	47,026	100.0%	6	8	13	24.2%
G34	Malate dehydrogenase, mitochondrial	IPI00323592	35,612	100.0%	8	9	16	43.8%
G35	Succinyl-CoA ligase [GDP-forming] subunit alpha, mitochondrial	IPI00406442	36,155	100.0%	3	3	6	11.6%
G36	Aldose reductase	IPI00223757	35,733	100.0%	10	12	19	31.6%
G37	Myosin light chain 3	IPI00133392	22,422	100.0%	2	2	3	20.6%
G37	Hydroxyacyl-coenzyme A dehydrogenase, mitochondrial	IPI00121105	34,465	100.0%	3	3	3	13.4%
G38	Hydroxyacyl-coenzyme A dehydrogenase, mitochondrial	IPI00121105	34,465	100.0%	5	5	7	13.7%
G39	Electron transfer flavoprotein subunit beta	IPI00121440	27,623	100.0%	14	18	31	55.7%
G40	Peroxiredoxin-1	IPI00121788	22,178	100.0%	7	8	15	33.2%
G41	ATP synthase subunit O, mitochondrial	IPI00118986	23,364	100.0%	4	5	12	22.1%
G42	Superoxide dismutase [Mn], mitochondrial	IPI00109109	24,603	100.0%	5	8	13	25.7%
G43	Alpha-crystallin B chain	IPI00138274	20,069	100.0%	6	7	14	29.7%
G44	Nucleoside diphosphate kinase B	IPI00127417	17,363	100.0%	6	10	17	40.1%
G45	Myoglobin	IPI00230760	17,070	100.0%	4	5	9	37.0%
G46	Myoglobin	IPI00230760	17,070	100.0%	11	14	29	60.4%
G47	Hemoglobin alpha, adult chain 2	IPI00845802	15,112	100.0%	6	9	16	62.0%
G48	Cytochrome c oxidase subunit 6B1	IPI00225390	10,072	100.0%	2	2	4	24.4%

Supplementary Table 3. Identifications of selected protein spots of adult zebrafish hearts.

Spot	Protein name	Accession Number	Mw (Da)	Protein identification probability	#unique peptides	#unique spectra	#total spectra	Percentage sequence coverage
R01	Cardiac myosin light chain 2	IPI00481849	19,021	100.0%	6	8	15	38.4%
R02	Cardiac myosin light chain 2	IPI00481849	19,021	100.0%	9	13	25	45.3%
R03	Cardiac myosin light chain 2	IPI00481849	19,021	100.0%	11	18	26	62.8%
R04	Cardiac myosin light chain 1	IPI00616566	21,865	100.0%	11	16	24	56.6%
R05	Cardiac myosin light chain 1	IPI00616566	21,865	100.0%	15	27	44	65.8%
R06	Unidentified	-	-	-	-	-	-	-
R07	Cytochrome c oxidase subunit Vaa	IPI00488350	15,897	100.0%	2	2	3	18.4%
R08	Fatty acid binding protein 3, muscle and heart	IPI00491514	14,882	100.0%	4	4	6	26.3%
R09	Histone H4	IPI00482809	11,368	100.0%	4	4	7	40.8%
R10	NADH dehydrogenase (Ubiquinone) Fe-S protein 3	IPI00503659	29,484	100.0%	2	4	7	9.8%
R11	Cardiac myosin light chain 1	IPI00616566	21,865	100.0%	3	4	6	14.8%
R12	Apolipoprotein A (apoa1b)	IPI00994508	30,140	100.0%	9	12	22	26.1%
R13	ATP synthase subunit beta	IPI00897805	56,476	100.0%	4	5	5	20.2%
R14	Heat shock 60kD protein 1 (Chaperonin)	IPI00508003	61,197	100.0%	14	16	24	24.0%
R15	Heat shock 60kD protein 1 (Chaperonin)	IPI00508003	61,197	100.0%	17	21	36	41.7%
R16	Uncharacterized protein	IPI00628054	72,714	100.0%	4	4	7	5.7%
R17	Gesolin A	IPI01016654	80,625	100.0%	8	10	20	19.2%
R18	NADH dehydrogenase (Ubiquinone) Fe-S protein 1	IPI00500466	79,433	100.0%	15	15	26	21.2%
R18	Ventricular myosin heavy chain-like protein	IPI00616127	224,172	100.0%	22	26	43	10.6%
R19	NADH dehydrogenase (Ubiquinone) Fe-S protein 1	IPI00500466	79,433	100.0%	19	23	41	24.8%
R19	Ventricular myosin heavy chain-like protein	IPI00616127	224,172	100.0%	13	16	24	7.8%
R20	NADH dehydrogenase (Ubiquinone) Fe-S protein 1	IPI00500466	79,433	100.0%	21	24	44	25.7%
R21	NADH dehydrogenase (Ubiquinone) Fe-S protein 1	IPI00500466	79,433	100.0%	3	3	5	4.1%
R22	Acetyltransferase component of pyruvate dehydrogenase complex	IPI00492140	69,208	100.0%	4	6	12	9.7%
R23	Aconitase 2, mitochondrial	IPI00483652	84,862	100.0%	8	9	17	19.3%
R24	Aconitase 2, mitochondrial	IPI00483652	84,862	100.0%	10	10	19	18.7%
R25	Aconitase 2, mitochondrial	IPI00483652	84,862	100.0%	13	15	27	26.5%
R26	Aconitase 2, mitochondrial	IPI00483652	84,862	100.0%	15	18	30	29.2%
R27	Transferrin	IPI00972722	73,533	100.0%	12	14	22	23.1%
R28	Unidentified	-	-	-	-	-	-	-
R29	Fibrinogen, B beta polypeptide	IPI00501169	54,408	100.0%	8	8	14	19.6%
R30	Fibrinogen, B beta polypeptide	IPI00501169	54,408	100.0%	16	20	34	37.5%
R31	Elongation factor 1-alpha	IPI00512240	50,048	100.0%	6	8	13	20.1%
R32	Enolase	IPI00483215	47,075	100.0%	8	9	17	27.3%
R32	beta-enolase	IPI00490877	47,473	100.0%	9	14	23	24.0%
R33	beta-enolase	IPI00490877	47,473	100.0%	14	22	38	37.4%
R34	Glyceraldehyde-3-phosphate dehydrogenase	IPI00497753	35,784	100.0%	8	11	21	32.4%
R35	Voltage-dependent anion channel 3	IPI00851926	32,854	100.0%	5	6	10	26.0%
R36	Malate dehydrogenase	IPI00495240	36,258	100.0%	7	12	23	22.8%
R37	Malate dehydrogenase	IPI00512157	35,420	100.0%	15	23	37	52.5%
R38	Four and a half LIM domains protein a	IPI00497720	39,095	100.0%	4	4	7	14.7%
R38	Malate dehydrogenase	IPI00512157	35,420	100.0%	4	4	7	13.4%
R38	ATP synthase subunit alpha	IPI00491975	59,745	100.0%	5	5	8	10.7%
R39	Myoglobin	IPI00490915	15,584	100.0%	8	11	19	76.2%
R40	Glutathione S-transferase pi	IPI00502668	23,528	100.0%	5	5	9	23.1%
R40	Glutathione S-transferase kappa 1	IPI00489672	25,908	100.0%	4	5	9	16.2%
R41	ATP synthase oligomycin sensitivity conferral protein	IPI00483070	22,483	100.0%	6	9	17	40.7%
R42	Superoxide dismutase	IPI00486525	25,008	100.0%	4	7	11	19.6%
R43	ATP synthase, H+ transporting, mitochondrial F0 complex, subunit d	IPI00484712	18,259	100.0%	8	14	23	36.0%
R44	Muscle cofilin 2	IPI00511345	18,771	100.0%	3	3	6	23.0%
R45	Nucleoside diphosphate kinase	IPI00497989	17,124	100.0%	7	14	21	53.6%
R46	Peptidyl-prolyl cis-trans isomerase	IPI00497774	17,489	100.0%	5	5	8	31.1%
R47	Myoglobin	IPI00490915	15,584	100.0%	18	27	49	85.0%
R48	Unidentified	-	-	-	-	-	-	-

Supplementary Table 4. Identifications of differentially expressed protein spots between cardiomyocytes from Day 0 and adult mice.

No.	Protein name	UniProt ID	pI	Mw (Da)	# Unique peptide	# Unique spectrum	# Total spectrum	Sequence coverage	Day 0 vs Adult	
									T-test	Av. Ratio
1	Calreticulin	CALR_MOUSE	4.33	46,347	11	12	20	27.90%	9.70E-07	-3.66
2	Tropomyosin alpha-1 chain	TPM1_MOUSE	4.69	32,681	5	5	9	18.30%	0.00012	-2.91
3	78 kDa glucose-regulated protein	GRP78_MOUSE	5.01	70,465	19	24	37	37.40%	4.30E-06	-1.89
4	Desmin	DESM_MOUSE	5.21	53,498	12	12	22	27.70%	8.20E-05	-2.84
4	Vimentin	VIME_MOUSE	5.05	53,556	14	16	23	41.20%	8.20E-05	-2.84
4	Myosin-6	MYH6_MOUSE	5.57	223,565	20	21	34	11.70%	8.20E-05	-2.84
5	ATP synthase subunit beta, mitochondrial	ATPB_MOUSE	4.99	51,749	13	18	29	36.30%	6.30E-05	-4.20
5	Myosin-6	MYH6_MOUSE	5.57	223,565	17	19	28	10.60%	6.30E-05	-4.20
6	Unidentified	-	-	-	-	-	-	-	0.0051	-3.17
7	Alpha-fetoprotein	FETA_MOUSE	5.54	65,343	20	24	41	38.50%	0.00023	-6.50
8	Alpha-fetoprotein	FETA_MOUSE	5.54	65,343	23	29	47	45.30%	0.0002	-7.71
8	Myosin-6	MYH6_MOUSE	5.57	223,565	23	28	48	10.70%	0.0002	-7.71
9	Heterogeneous nuclear ribonucleoprotein K	HNRPK_MOUSE	5.39	50,976	10	11	17	27.40%	0.03	-2.55
9	Desmin	DESM_MOUSE	5.21	53,498	14	14	23	32.00%	0.03	-2.55
9	Myosin-6	MYH6_MOUSE	5.57	223,565	15	15	24	8.36%	0.03	-2.55
10	Alpha-fetoprotein	FETA_MOUSE	5.54	65,343	20	28	47	44.80%	0.00054	-6.30
10	Myosin-6	MYH6_MOUSE	5.57	223,565	22	26	43	11.10%	0.00054	-6.30
11	Gelsolin	GELS_MOUSE	5.72	83,344	14	15	23	23.30%	0.0011	-4.19
11	Myosin-6	MYH6_MOUSE	5.57	223,565	17	18	27	10.40%	0.0011	-4.19
12	Gelsolin	GELS_MOUSE	5.72	83,344	22	27	44	36.80%	0.00036	-6.01
13	Dihydrolipoyllysine-residue acetyltransferase component of pyruvate dehydrogenase complex, mitochondrial	ODP2_MOUSE	5.70	58,778	14	22	35	29.10%	0.0081	1.75
14	Aconitate hydratase, mitochondrial	ACON_MOUSE	8.08	85,464	12	12	17	19.00%	0.00023	1.75
15	Unidentified	-	-	-	-	-	-	-	9.20E-06	-3.36
16	Tubulin beta-4B chain	TBB4B_MOUSE	4.79	49,831	18	22	33	58.20%	6.50E-05	-3.14
17	ATP synthase subunit beta, mitochondrial	ATPB_MOUSE	4.99	51,749	16	29	55	41.40%	5.70E-06	3.39
18	Protein disulfide-isomerase A3	PDIA3_MOUSE	5.69	54,267	13	14	18	28.10%	1.40E-05	-2.91
19	T-complex protein 1 subunit epsilon	TCPE_MOUSE	5.72	59,493	6	7	10	14.00%	6.30E-06	-2.95
20	T-complex protein 1 subunit epsilon	TCPE_MOUSE	5.72	59,493	16	19	30	37.90%	4.20E-05	-3.18
21	Unidentified	-	-	-	-	-	-	-	7.30E-06	-3.69
22	Adenylyl cyclase-associated protein 2	CAP2_MOUSE	6.00	52,862	9	9	13	20.20%	0.00082	-2.15
22	Bifunctional epoxide hydrolase 2	HYES_MOUSE	5.85	62,515	11	13	23	22.00%	0.00082	-2.15
23	Unidentified	-	-	-	-	-	-	-	0.00039	-3.33
24	Dihydropyrimidinase-related protein 3	DPYL3_MOUSE	6.04	61,936	8	8	13	20.20%	4.30E-05	-3.69
25	Elongation factor Tu, mitochondrial	EFTU_MOUSE	6.20	44,971	18	27	42	51.80%	9.80E-05	-2.89
26	Hydroxysteroid dehydrogenase-like protein 2	HSDL2_MOUSE	6.31	54,208	10	11	14	23.30%	0.01	-2.61
26	T-complex protein 1 subunit gamma	TCPG_MOUSE	6.28	60,630	10	10	15	26.10%	0.01	-2.61
26	Stress-induced-phosphoprotein 1	STIP1_MOUSE	6.40	62,582	12	12	14	26.00%	0.01	-2.61
27	Aldehyde dehydrogenase X, mitochondrial	AL1B1_MOUSE	6.13	55,401	13	15	24	28.10%	0.0019	-3.13
28	Aldehyde dehydrogenase X, mitochondrial	AL1B1_MOUSE	6.13	55,401	5	6	12	10.60%	0.00014	-4.75
29	ATP synthase subunit alpha, mitochondrial	ATPA_MOUSE	8.28	55,310	18	22	39	40.50%	3.90E-05	2.94
30	Actin, alpha cardiac muscle 1	ACTC_MOUSE	5.23	41,785	12	18	46	34.70%	0.019	1.24
31	Troponin T, cardiac muscle	TNNT2_MOUSE	4.98	35,694	12	17	28	32.20%	9.20E-07	3.60
32	Creatine kinase B-type	KCRB_MOUSE	5.40	42,582	12	16	26	42.00%	0.00031	-3.16
33	Alpha-enolase	ENOA_MOUSE	6.36	47,010	14	17	31	35.90%	6.80E-06	-3.89
34	Alpha-enolase	ENOA_MOUSE	6.36	47,010	18	24	40	49.50%	5.20E-05	-4.12
35	Alpha-enolase	ENOA_MOUSE	6.36	47,010	18	30	48	46.50%	1.40E-05	-4.20
36	Pyruvate dehydrogenase E1 component subunit alpha, somatic form, mitochondrial	ODPA_MOUSE	6.78	40,181	5	6	8	14.40%	0.0077	-2.01
36	Alpha-centractin	ACTZ_MOUSE	6.19	42,614	6	7	11	23.90%	0.0077	-2.01
37	Fumarate hydratase, mitochondrial	FUMH_MOUSE	7.88	49,921	13	14	19	39.60%	1.30E-05	2.81

38	3-ketoacyl-CoA thiolase, mitochondrial	THIM_MOUSE	8.33	41,830	8	11	20	32.70%	4.40E-05	4.40
38	Creatine kinase S-type, mitochondrial	KCRS_MOUSE	7.72	43,387	11	14	20	32.00%	4.40E-05	4.40
38	Medium-chain specific acyl-CoA dehydrogenase, mitochondrial	ACADM_MOUSE	7.69	43,593	9	12	21	24.20%	4.40E-05	4.40
38	Phosphoglycerate kinase 1	PGK1_MOUSE	8.02	44,419	11	17	29	37.20%	4.40E-05	4.40
39	3-ketoacyl-CoA thiolase, mitochondrial	THIM_MOUSE	8.33	41,830	12	17	26	52.10%	0.0011	4.18
40	3-ketoacyl-CoA thiolase, mitochondrial	THIM_MOUSE	8.33	41,830	12	16	26	49.40%	0.00023	5.56
40	Creatine kinase S-type, mitochondrial	KCRS_MOUSE	7.72	43,387	13	19	29	39.90%	0.00023	5.56
40	Phosphoglycerate kinase 1	PGK1_MOUSE	8.02	44,419	17	24	36	49.40%	0.00023	5.56
41	3-ketoacyl-CoA thiolase, mitochondrial	THIM_MOUSE	8.33	41,830	21	33	46	73.80%	0.0013	2.86
42	Tropomyosin alpha-1 chain	TPM1_MOUSE	4.69	32,681	21	32	58	54.20%	2.40E-05	2.03
43	Elongation factor 1-delta	EF1D_MOUSE	4.91	31,162	9	10	18	37.70%	0.0001	-2.68
44	Pyruvate dehydrogenase E1 component subunit beta, mitochondrial	ODPB_MOUSE	5.39	35,768	12	17	33	44.80%	0.00023	2.14
46	Isocitrate dehydrogenase [NAD] subunit alpha, mitochondrial	IDH3A_MOUSE	5.59	36,707	2	3	5	6.83%	3.20E-05	2.79
46	Isocitrate dehydrogenase [NAD] subunit alpha, mitochondrial	IDH3A_MOUSE	5.59	36,707	11	13	19	35.20%	0.011	1.24
47	Malate dehydrogenase, cytoplasmic	MDHC_MOUSE	6.16	36,380	10	12	27	35.90%	0.00025	1.59
48	Delta(3,5)-Delta(2,4)-dienoyl-CoA isomerase, mitochondrial	ECH1_MOUSE	6.01	32,433	8	12	23	37.00%	4.10E-06	5.39
49	Myozenin-2	MYOZ2_MOUSE	8.53	29,762	5	5	9	20.80%	2.40E-05	4.64
49	Four and a half LIM domains protein 2	FHL2_MOUSE	7.31	32,073	5	5	6	20.80%	2.40E-05	4.64
49	Malate dehydrogenase, cytoplasmic	MDHC_MOUSE	6.16	36,380	4	4	7	14.40%	2.40E-05	4.64
50	Unidentified	-	-	-	-	-	-	-	0.0001	4.22
51	Electron transfer flavoprotein subunit alpha, mitochondrial	ETFA_MOUSE	8.62	35,009	8	14	25	35.70%	8.20E-07	4.64
52	Aldose reductase	ALDR_MOUSE	6.79	35,601	9	10	14	32.00%	0.029	-2.08
52	Annexin A2	ANXA2_MOUSE	7.53	38,545	13	15	23	38.30%	0.029	-2.08
53	Malate dehydrogenase, mitochondrial	MDHM_MOUSE	8.55	33,139	10	10	15	39.60%	9.80E-06	-1.79
53	Glyceraldehyde-3-phosphate dehydrogenase	G3P_MOUSE	8.45	35,679	9	11	19	30.30%	9.80E-06	-1.79
53	Annexin A2	ANXA2_MOUSE	7.53	38,545	12	14	20	42.80%	9.80E-06	-1.79
54	Electron transfer flavoprotein subunit alpha, mitochondrial	ETFA_MOUSE	8.62	35,009	8	12	19	32.70%	7.20E-07	3.09
55	Voltage-dependent anion-selective channel protein 1	VDAC1_MOUSE	8.55	32,351	11	14	21	48.30%	0.0081	2.01
56	Carbonyl reductase [NADPH] 1	CBR1_MOUSE	8.53	30,510	6	6	7	24.90%	0.0074	1.50
56	Voltage-dependent anion-selective channel protein 1	VDAC1_MOUSE	8.55	32,351	5	5	8	24.70%	0.0074	1.50
57	2,4-dienoyl-CoA reductase, mitochondrial	DECR_MOUSE	8.78	32,451	5	5	9	20.00%	0.012	1.87
58	Voltage-dependent anion-selective channel protein 1	VDAC1_MOUSE	8.55	32,351	8	9	16	38.90%	6.00E-05	2.87
59	14-3-3 protein epsilon	1433E_MOUSE	4.63	29,174	8	10	13	36.10%	1.40E-06	-2.94
60	Complement component 1 Q subcomponent-binding protein, mitochondrial	C1QBP_MOUSE	4.41	23,766	5	8	11	24.80%	0.00015	-2.66
61	Electron transfer flavoprotein subunit beta	ETFB_MOUSE	8.29	27,492	11	18	29	43.10%	0.0012	-2.92
62	Apolipoprotein A-I	APOA1_MOUSE	5.31	27,950	9	11	18	36.70%	3.10E-05	-2.70
63	Microtubule-associated protein RP/EB family member 1	MARE1_MOUSE	5.12	29,885	3	3	5	14.60%	0.00044	-2.30
63	Ubiquinone biosynthesis protein COQ9, mitochondrial	COQ9_MOUSE	4.93	30,241	3	3	5	12.80%	0.00044	-2.30
64	Apolipoprotein A-I	APOA1_MOUSE	5.31	27,950	7	9	14	29.20%	1.30E-05	-4.06
64	Microtubule-associated protein RP/EB family member 1	MARE1_MOUSE	5.12	29,885	7	9	18	36.20%	1.30E-05	-4.06
65	Chloride intracellular channel protein 1	CLIC1_MOUSE	5.09	26,882	10	12	18	52.30%	0.00015	-3.84
66	Carbonic anhydrase 2	CAH2_MOUSE	6.48	28,901	5	6	11	24.60%	0.01	1.50
66	Bisphosphoglycerate mutase	PMGE_MOUSE	6.56	29,847	5	5	8	24.30%	0.01	1.50
67	Phosphoglycerate mutase 1	PGAM1_MOUSE	6.75	28,701	10	17	26	50.00%	1.60E-07	-3.49
68	Adenylate kinase 2, mitochondrial	KAD2_MOUSE	6.96	26,469	5	6	9	24.70%	0.013	1.16
68	Phosphoglycerate mutase 1	PGAM1_MOUSE	6.75	28,701	5	6	8	33.50%	0.013	1.16
69	Adenylate kinase 2, mitochondrial	KAD2_MOUSE	6.96	26,469	5	6	10	28.00%	0.00053	1.71
69	Electron transfer flavoprotein subunit beta	ETFB_MOUSE	8.29	27,492	8	8	11	31.40%	0.00053	1.71
69	Enoyl-CoA hydratase, mitochondrial	ECHM_MOUSE	7.78	28,475	4	5	8	19.70%	0.00053	1.71
70	Enoyl-CoA hydratase, mitochondrial	ECHM_MOUSE	7.78	28,475	9	11	18	30.00%	0.0021	1.86
71	Electron transfer flavoprotein subunit beta	ETFB_MOUSE	8.29	27,492	6	6	10	26.30%	5.10E-06	2.00
71	Enoyl-CoA hydratase, mitochondrial	ECHM_MOUSE	7.78	28,475	6	7	12	19.70%	5.10E-06	2.00
72	Ubiquitin-conjugating enzyme E2 R1	UB2R1_MOUSE	4.44	26,622	2	2	4	9.36%	5.60E-06	2.31
73	Electron transfer flavoprotein subunit beta	ETFB_MOUSE	8.29	27,492	9	12	22	41.60%	2.70E-06	2.73

73	Succinate dehydrogenase [ubiquinone] iron-sulfur subunit, mitochondrial	DHSB_MOUSE	8.69	28,770	6	8	12	28.00%	2.70E-06	2.73
73	Phosphoglycerate mutase 2	PGAM2_MOUSE	8.65	28,827	8	10	16	30.40%	2.70E-06	2.73
73	Enoyl-CoA delta isomerase 1, mitochondrial	ECI1_MOUSE	8.23	29,224	6	9	15	26.00%	2.70E-06	2.73
74	Coiled-coil-helix-coiled-coil-helix domain-containing protein 3, mitochondrial	CHCH3_MOUSE	8.57	26,203	8	9	13	25.60%	4.60E-05	1.77
74	Proteasome subunit alpha type-7	PSA7_MOUSE	8.59	27,855	6	7	11	34.30%	4.60E-05	1.77
74	Succinate dehydrogenase [ubiquinone] iron-sulfur subunit, mitochondrial	DHSB_MOUSE	8.69	28,770	10	13	16	43.60%	4.60E-05	1.77
75	Succinate dehydrogenase [ubiquinone] iron-sulfur subunit, mitochondrial	DHSB_MOUSE	8.69	28,770	11	16	28	43.60%	0.011	1.39
75	Phosphoglycerate mutase 2	PGAM2_MOUSE	8.65	28,827	9	12	20	30.80%	0.011	1.39
76	Translationally-controlled tumor protein	TCTP_MOUSE	4.76	19,462	6	9	14	34.90%	1.60E-05	-2.42
77	Translationally-controlled tumor protein	TCTP_MOUSE	4.76	19,462	7	10	19	41.90%	7.80E-06	-2.59
78	Myosin light chain 3	MYL3_MOUSE	5.03	22,290	8	13	23	52.50%	6.40E-06	1.94
79	Myosin light chain 3	MYL3_MOUSE	5.03	22,290	13	21	53	70.60%	1.60E-05	1.69
80	Myosin light chain 3	MYL3_MOUSE	5.03	22,290	10	13	22	57.40%	0.00042	1.59
81	NADH dehydrogenase [ubiquinone] iron-sulfur protein 8, mitochondrial	NDUS8_MOUSE	5.13	20,442	4	6	11	25.90%	0.0013	1.93
82	Osteoclast-stimulating factor 1	OSTF1_MOUSE	5.44	23,783	2	2	3	9.30%	0.001	-2.42
82	Pre-mRNA-splicing factor SPF27	SPF27_MOUSE	5.48	26,000	3	3	3	13.80%	0.001	-2.42
82	NADH dehydrogenase [ubiquinone] iron-sulfur protein 3, mitochondrial	NDUS3_MOUSE	5.45	26,479	3	3	3	14.10%	0.001	-2.42
83	Chloride intracellular channel protein 4	CLIC4_MOUSE	5.45	28,598	12	16	24	52.60%	1.50E-06	-3.88
84	Serum amyloid P-component	SAMP_MOUSE	5.97	26,247	6	8	15	24.10%	0.0049	-3.28
85	NADH dehydrogenase [ubiquinone] flavoprotein 2, mitochondrial	NDUV2_MOUSE	5.31	23,847	11	20	33	55.60%	1.40E-05	1.77
85	Apolipoprotein A-I	APOA1_MOUSE	5.31	27,950	14	20	34	45.80%	1.40E-05	1.77
86	Thioredoxin-dependent peroxide reductase, mitochondrial	PRDX3_MOUSE	5.73	21,565	6	8	13	28.00%	1.10E-05	2.22
86	BAG family molecular chaperone regulator 2	BAG2_MOUSE	6.01	23,474	5	5	6	24.30%	1.10E-05	2.22
87	Peroxiredoxin-4	PRDX4_MOUSE	5.83	26,476	8	8	13	45.30%	6.50E-05	-5.27
88	28 kDa heat- and acid-stable phosphoprotein	HAP28_MOUSE	6.80	20,605	3	3	5	19.30%	1.20E-05	-2.14
88	Heat shock protein beta-1	HSPB1_MOUSE	6.12	23,014	5	5	8	24.90%	1.20E-05	-2.14
89	Alpha-crystallin B chain	CRYAB_MOUSE	6.76	20,069	7	7	10	46.90%	1.30E-05	1.99
90	ES1 protein homolog, mitochondrial	ES1_MOUSE	7.31	23,920	9	16	29	44.00%	6.80E-06	3.11
90	Glutathione S-transferase Mu 2	GSTM2_MOUSE	7.31	25,586	10	13	22	44.50%	6.80E-06	3.11
91	NADH dehydrogenase [ubiquinone] 1 beta subcomplex subunit 9	NDUB9_MOUSE	7.84	21,853	3	5	8	21.20%	1.40E-06	2.76
91	Glutathione S-transferase P 1	GSTP1_MOUSE	8.13	23,478	5	6	14	31.90%	1.40E-06	2.76
92	Peroxiredoxin-1	PRDX1_MOUSE	8.26	22,045	7	9	15	45.70%	4.80E-06	2.91
93	Glutathione S-transferase Mu 1	GSTM1_MOUSE	8.14	25,839	6	9	16	40.40%	0.00044	2.78
94	NADH dehydrogenase [ubiquinone] 1 beta subcomplex subunit 10	NDUBA_MOUSE	8.19	21,024	8	11	17	46.00%	0.00018	1.95
94	Glutathione S-transferase P 1	GSTP1_MOUSE	8.13	23,478	8	9	16	48.10%	0.00018	1.95
95	Protein NipSnap homolog 2	NIPS2_MOUSE	9.31	32,933	3	5	9	11.70%	3.40E-05	2.06
96	GTP:AMP phosphotransferase AK3, mitochondrial	KAD3_MOUSE	8.88	25,295	3	3	6	16.70%	0.0029	1.76
96	Protein NipSnap homolog 2	NIPS2_MOUSE	9.31	32,933	4	4	8	17.40%	0.0029	1.76
97	Myosin light chain 4	MYL4_MOUSE	4.96	21,159	8	11	17	50.30%	0.001	-2.20
98	Myosin light chain 4	MYL4_MOUSE	4.96	21,159	5	9	15	32.10%	0.033	-1.26
99	Myosin light chain 4	MYL4_MOUSE	4.96	21,159	6	7	10	36.80%	0.00038	-1.74
99	Peroxiredoxin-2	PRDX2_MOUSE	5.20	21,647	7	8	13	49.00%	0.00038	-1.74
100	Chromobox protein homolog 3	CBX3_MOUSE	5.13	20,855	6	8	14	36.60%	0.00072	-3.55
101	39S ribosomal protein L12, mitochondrial	RM12_MOUSE	5.35	16,653	4	4	7	24.40%	5.30E-05	-3.13
102	ATP synthase subunit d, mitochondrial	ATP5H_MOUSE	5.53	18,618	8	8	15	53.40%	8.60E-07	3.32
103	Adenylate kinase isoenzyme 1	KAD1_MOUSE	5.67	21,540	8	9	17	50.00%	1.10E-05	2.29
104	ATP synthase subunit d, mitochondrial	ATP5H_MOUSE	5.53	18,618	2	2	2	18.00%	0.0038	2.17
105	ATP synthase subunit d, mitochondrial	ATP5H_MOUSE	5.53	18,618	14	20	36	80.70%	2.70E-06	2.19
106	Ferritin heavy chain	FRIH_MOUSE	5.53	21,067	4	5	8	27.50%	0.0015	1.56
107	Putative hydrolase RBBP9	RBBP9_MOUSE	5.62	20,912	5	6	10	26.90%	0.0034	-2.53
108	Ferritin light chain 1	FRIL1_MOUSE	5.65	20,802	9	14	26	61.20%	0.011	-1.85
109	Coactosin-like protein	COTL1_MOUSE	5.23	15,813	1	1	1	5.63%	0.00015	-2.96
110	Tubulin-specific chaperone A	TBCA_MOUSE	5.25	12,626	6	9	14	38.90%	0.0077	-2.04
111	Programmed cell death protein 5	PDCD5_MOUSE	5.56	14,144	4	6	10	37.30%	1.70E-06	-3.30

112	Retinol-binding protein 1	RET1_MOUSE	5.11	15,715	8	12	22	54.80%	6.50E-06	-3.23
113	Retinol-binding protein 1	RET1_MOUSE	5.11	15,715	6	8	10	47.40%	0.0018	2.40
114	Cytochrome c oxidase subunit 5A, mitochondrial	COX5A_MOUSE	5.01	12,436	5	6	12	43.20%	6.70E-06	2.30
115	Cytochrome c oxidase subunit 5A, mitochondrial	COX5A_MOUSE	5.01	12,436	3	4	8	21.90%	0.0011	2.29
116	Unidentified	-	- -	-	-	-	-		1.20E-06	2.90
117	Fatty acid-binding protein, heart	FABPH_MOUSE	6.15	14,688	7	11	16	55.60%	3.80E-07	6.93
118	Fatty acid-binding protein, heart	FABPH_MOUSE	6.15	14,688	2	3	4	21.10%	0.00072	2.67
119	Costars family protein ABRACL	ABRAL_MOUSE	5.49	9,030	1	1	2	11.10%	5.60E-05	2.66
120	Fatty acid-binding protein, epidermal	FABP5_MOUSE	6.18	15,006	2	2	4	25.20%	0.004	-2.82
121	Fatty acid-binding protein, heart	FABPH_MOUSE	6.15	14,688	4	4	7	28.60%	1.30E-07	6.74
122	Fatty acid-binding protein, heart	FABPH_MOUSE	6.15	14,688	4	5	7	31.60%	0.0054	4.31
123	Fatty acid-binding protein, heart	FABPH_MOUSE	6.15	14,688	11	16	30	72.20%	7.00E-08	6.94
124	Enhancer of rudimentary homolog	ERH_MOUSE	5.62	12,128	2	4	7	26.90%	3.90E-08	-5.03
125	Cytochrome c oxidase subunit 5B, mitochondrial	COX5B_MOUSE	5.74	10,718	3	4	7	24.20%	1.40E-06	2.89
126	Cytochrome c oxidase subunit 5B, mitochondrial	COX5B_MOUSE	5.74	10,718	5	6	8	29.70%	8.70E-06	2.74
127	Stathmin	STMN1_MOUSE	5.76	17,143	7	10	17	32.20%	1.80E-09	-23.63
128	Heat shock protein beta-7	HSPB7_MOUSE	5.95	18,635	5	7	13	37.30%	0.0014	-1.92
129	Ubiquitin-conjugating enzyme E2 N	UBE2N_MOUSE	6.13	17,138	5	6	10	40.80%	0.00013	-2.02
130	Fatty acid-binding protein, epidermal	FABP5_MOUSE	6.18	15,006	2	2	2	14.80%	4.70E-05	-4.33
131	Fatty acid-binding protein, epidermal	FABP5_MOUSE	6.18	15,006	7	10	17	73.30%	4.10E-07	-6.69
132	Transthyretin	TTHY_MOUSE	5.77	15,776	5	7	14	28.60%	0.0078	-1.61
133	40S ribosomal protein S12	RS12_MOUSE	7.02	14,394	2	2	2	20.50%	6.60E-05	-2.36
134	40S ribosomal protein S12	RS12_MOUSE	7.02	14,394	3	4	7	22.00%	5.60E-06	-4.18
135	Nucleoside diphosphate kinase B	NDKB_MOUSE	7.17	17,232	6	9	15	46.10%	0.00042	5.60
136	Peptidyl-prolyl cis-trans isomerase A	PPIA_MOUSE	7.73	17,971	11	17	31	74.40%	0.05	-1.73
137	Myoglobin	MYG_MOUSE	7.23	16,938	6	9	12	48.70%	0.0003	3.06
138	Unidentified	-	- -	-	-	-	-		2.20E-05	2.90
139	Cofilin-1	COF1_MOUSE	8.26	18,428	8	9	13	47.60%	1.80E-05	-4.65
140	Peptidyl-prolyl cis-trans isomerase A	PPIA_MOUSE	7.73	17,971	10	16	27	66.50%	4.20E-05	5.86
141	NADH dehydrogenase [ubiquinone] 1 beta subcomplex subunit 7	NDUB7_MOUSE	8.38	16,200	5	5	9	44.50%	0.0032	-1.88
141	Destrin	DEST_MOUSE	8.19	18,390	6	7	10	40.60%	0.0032	-1.88
142	Myoglobin	MYG_MOUSE	7.23	16,938	10	16	30	65.60%	6.50E-06	9.75
143	Peroxiredoxin-5, mitochondrial	PRDX5_MOUSE	7.70	17,015	10	12	20	49.00%	2.90E-05	3.49
144	Myoglobin	MYG_MOUSE	7.23	16,938	7	10	14	61.70%	0.00027	4.38
145	Unidentified	-	- -	-	-	-	-		5.10E-05	2.19
146	Unidentified	-	- -	-	-	-	-		2.40E-07	9.14
147	Protein S100-A13	S10AD_MOUSE	5.89	11,158	2	2	4	20.40%	0.00031	5.02
148	ATP synthase-coupling factor 6, mitochondrial	ATP5J_MOUSE	5.40	8,945	4	5	7	40.70%	1.60E-05	4.73
149	NADH dehydrogenase [ubiquinone] iron-sulfur protein 6, mitochondrial	NDUS6_MOUSE	8.86	13,020	7	10	18	65.50%	0.023	2.16
150	Acylphosphatase-2	ACYP2_MOUSE	9.30	11,878	5	5	6	34.00%	0.00075	2.65
151	Cytochrome c oxidase subunit 6B1	CX6B1_MOUSE	8.97	9,940	7	7	12	66.30%	0.0049	3.17

Supplementary Figure 1. DIGE Analysis. **A)** Differentially expressed proteins between the adult mouse heart versus zebrafish (ZF) heart (**Supplementary Tables 2** and **3**, respectively). **B)** Differentially expressed proteins between the neonatal and adult mouse heart. Note that in panel B results of **Fig. 1B** were reproduced with different biological replicates using a dye swap. The numbered spots were analysed by LC-MS/MS.

A

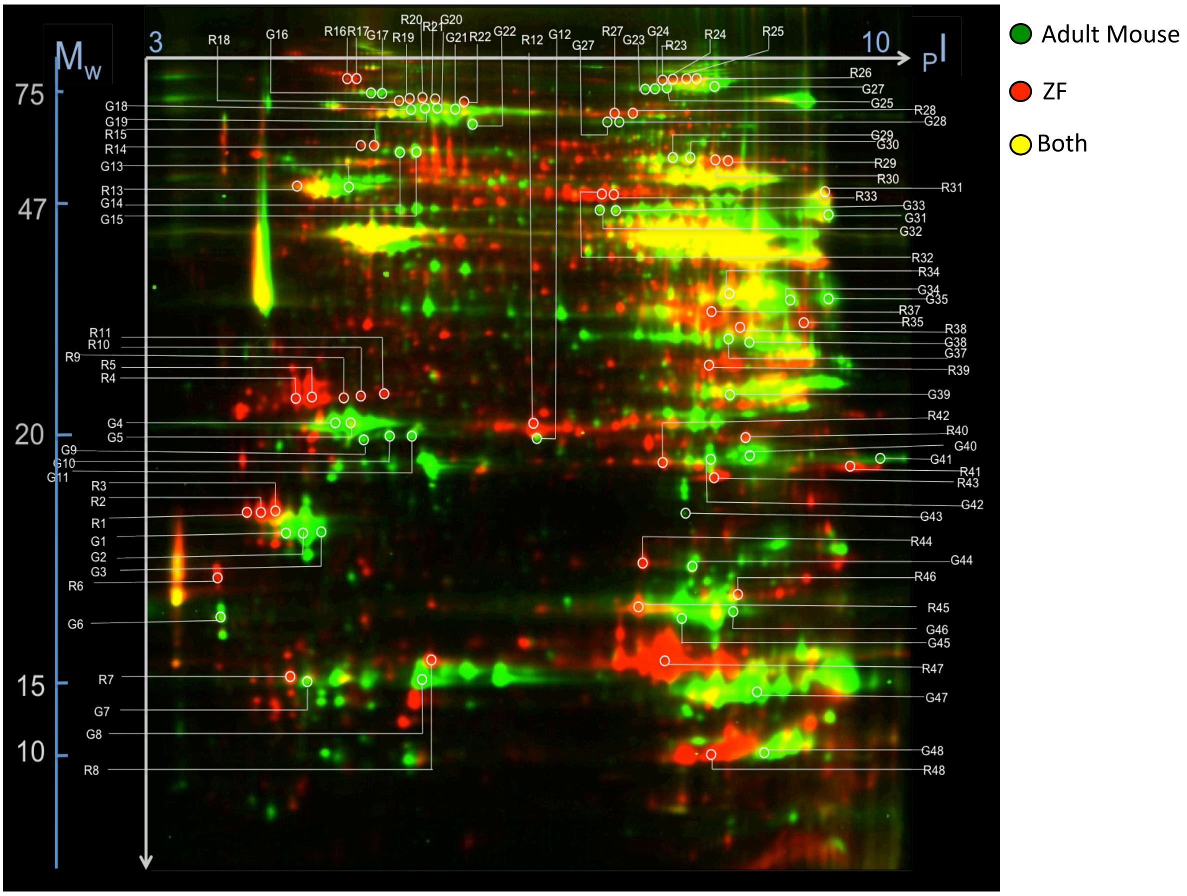

B

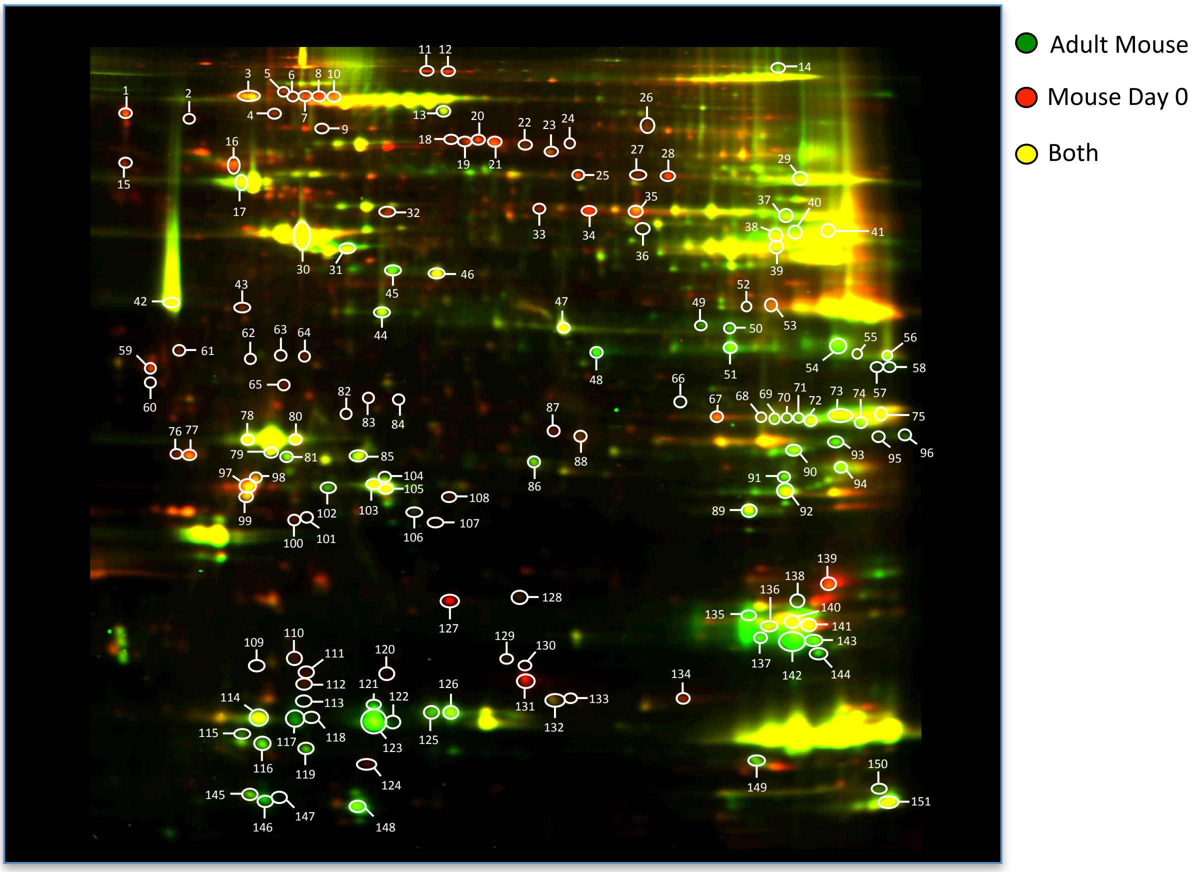

Supplementary Figure 2. Enrichment Analysis. Enrichment for biological processes, cellular localisation and molecular function for the proteins identified by DIGE, in adult mice and adult zebrafish (ZF) hearts (**Supplementary Tables 2 and 3**).

Adult Mouse Heart

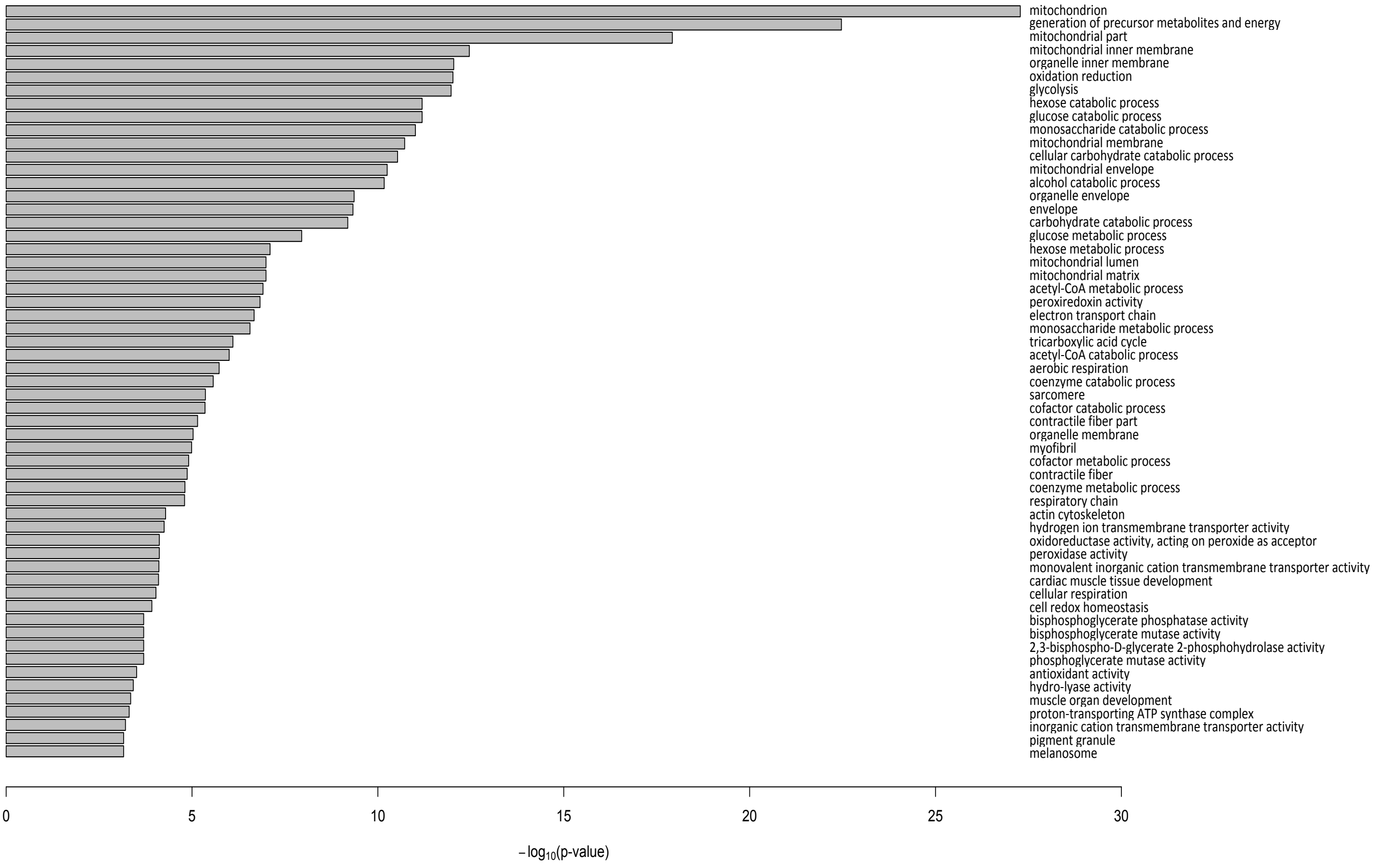

Adult ZF Heart

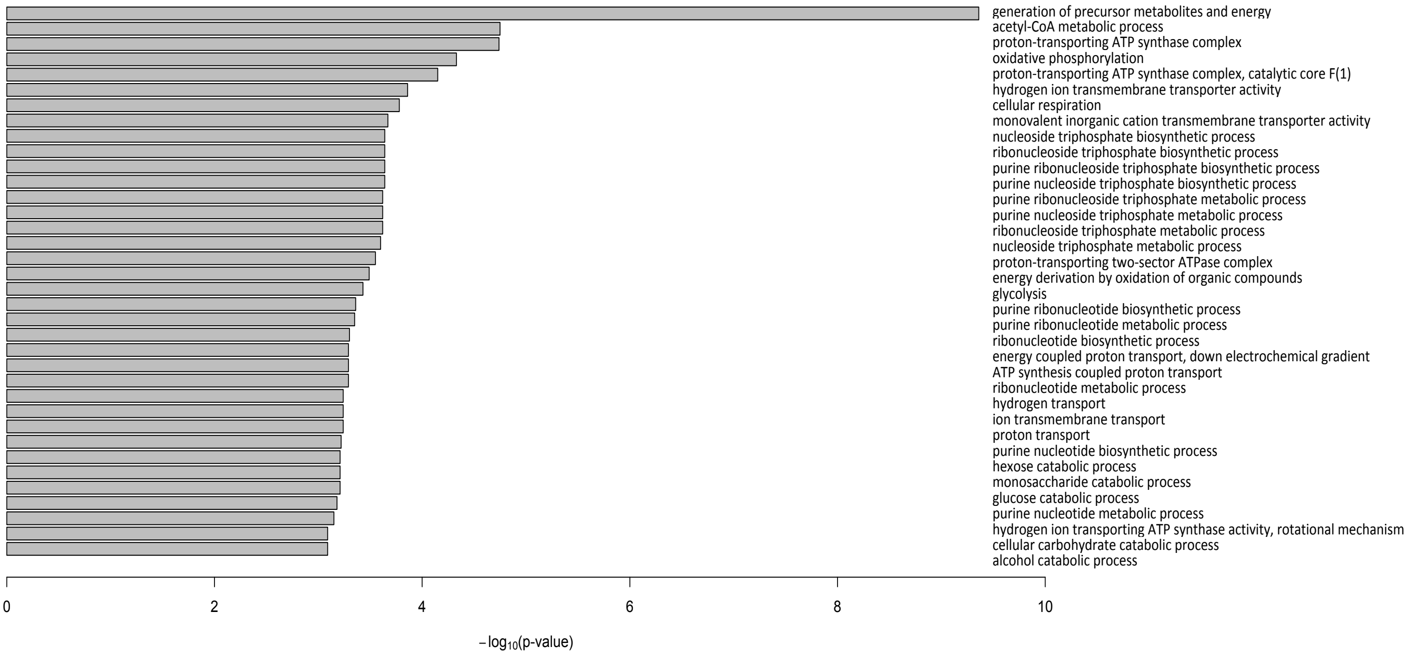

Supplementary Figure 3. Western Blot Analysis. **A)** Myozenin, nebulette and troponin T were proteins of interest in the DIGE analysis. Further immunoblot analyses were performed to corroborate the proteomics data. However, most antibodies are not validated for zebrafish. Thus, we cannot determine if the protein is absent or simply not recognised by the antibody. Note the different troponin T isoform in zebrafish at 25kDa. **B)** Densitometry quantitation. ** P<0.01, *** P<0.001. ZF, denotes "adult zebrafish".

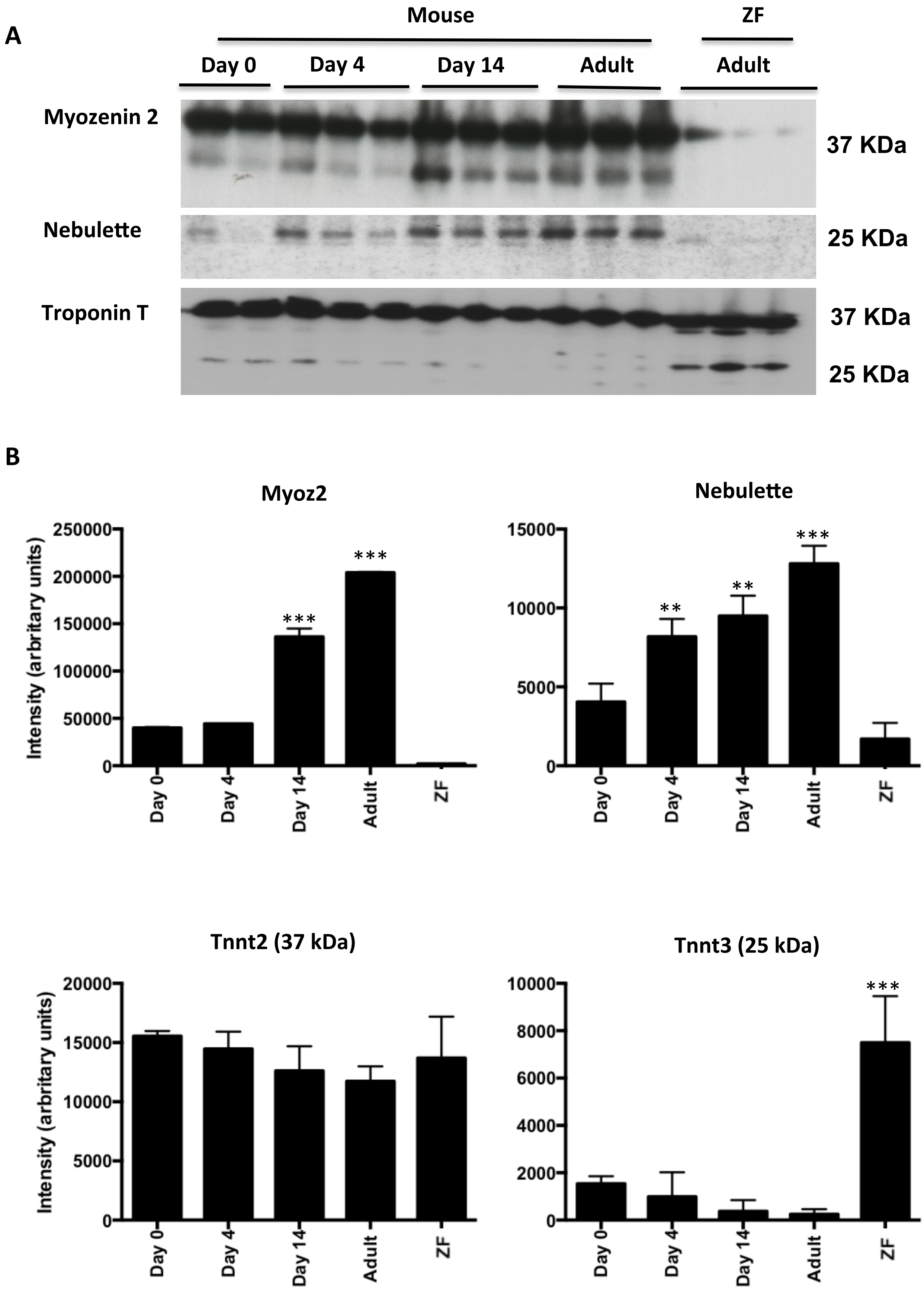

Supplementary Figure 4. qPCR Analysis. In order to compare gene expression between two species in a given gene family, a gene from that family that did not change significantly over time was used as the normalisation control as indicated. For myoz1 and myoz2, 18S RNA was used as an endogenous control. The relative abundance was ranked from lowest to highest expression (+, ++, +++) in neonatal mouse, adult mouse and adult zebrafish (ZF) hearts. The absence of expression or very low levels was denoted by (-). Summary in **Fig. 2A.**

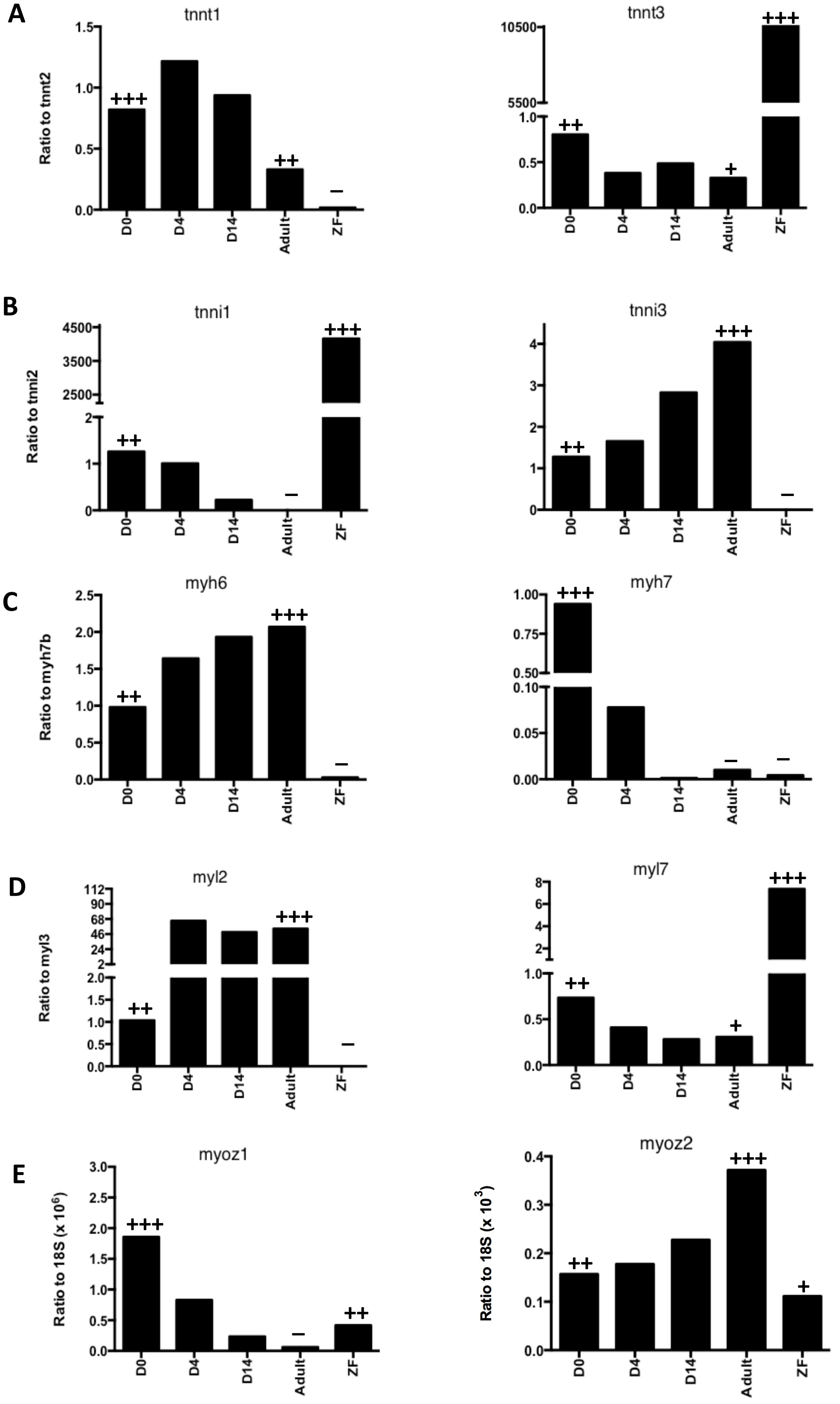

Supplement: Supplementary file 1 — Supplementary material. [file mmc1.pdf]
